# ICD-11 Personality Disorder: The Indispensable Turn to Narrative Identity

**DOI:** 10.3389/fpsyt.2021.642696

**Published:** 2021-02-18

**Authors:** Majse Lind

**Affiliations:** Department of Psychology, University of Florida, Gainesville, FL, United States

**Keywords:** ICD-11, narrative identity, autobiographical reasoning, personality disorder, treatment

## ICD-11 Personality Disorder: The Indispensable Narrative Turn

The field of personality disorders (PD) is taking an important step away from a categorical approach by inviting a dimensional understanding of conceptualizing and diagnosing manifestations of personality pathology (1). This means that, in the long awaited 11th revision of the International Classification of Diseases (ICD-11), the core of PD is considered on a spectrum of intra- and interpersonal dysfunctions (2). Let me begin this paper by declaring my sympathy for the dimensional model. I do, however, encourage this shift to incorporate an aspect of (maladaptive) personality that is gaining momentum in PD research—a person's *narrative identity*. In the following, I illustrate why narrative identity contributes with an indispensable aspect to ICD-11's self-function that revolves around “stability and coherence of one's sense of identity” (1). For additional and more thorough arguments on this matter see also Lind, Dunlop and Sharp (under review).

## Narrative Identity: The Temporally Coherent Self

Narrative identity is the internal and dynamic story of a person's life (3). As individuals are living unique lives, each has a unique story to tell. Narrative identity (i.e., the self as author) constitutes the most unique aspect of a person's personality (4) and conveys the innermost perspective of personhood relative to a shared-by-many trait profile (i.e., the self as actor) and general social-cognitive abilities [i.e., the self as agent; (5)].

Narrative identity helps us understand and express to others who we once were, who we are today, and who we may become in the future (5) and therefore affirms us with a sense of self-continuity (i.e., *coherence*) and *stability* that, through time and place, we continue to stay the same person (4). The emergence of a coherent narrative identity occurs in adolescence, as these individuals are faced with the developmental task to form a mature identity (6). Narrative identity emerges as a final, organizing element of personality after traits and social-cognitive abilities are established (3).

The emergence of *Autobiographical Reasoning* is a crucial cognitive tool conveying the competence to construct global coherence in the story (7). Autobiographical reasoning is the underlying reflective process in which past events are interpreted, organized, and evaluated to construct temporally, culturally, causally, and thematically coherent accounts of a person's life (8). Temporal coherence refers to the understanding of how events are timely related in the life story and is, at least in Western countries, typically expressed as a chronological order of events (8). Cultural coherence refers to the ability to integrate normative cultural life events in the story [e.g., getting a job and having children; (9)]. Causal coherence encompasses the ability to create meaningful connections between events and periods across the life story and how events have contributed to both change and/or stability within the person. Finally, thematic coherence relates to the ability to establish thematic links across a person's life. While causal- and thematic coherence are more sophisticated processes of coherence beginning to emerge in mid to late adolescence, autobiographical reasoning related to temporal and cultural coherence has an earlier starting point (7, 8).

## Narrative Identity as a Vital Self-Function Within ICD-11

Autobiographical reasoning provides nuance to conceptualizing and assessing “stability and coherence of one's sense of identity” within ICD-11 (1) by tackling coherence in terms of at least four distinct dimensions (i.e., chronological, cultural, causal, and thematic), while also providing an indicator of *global coherence* (8). With diminished autobiographical reasoning, a person's narrative identity appears disorganized and fragmented. A recent systematic review [see (10)] based on the last decade's studies on PD and narrative identity indicates that a disturbed autobiographical reasoning in people manifesting PD could play a key role in inducing a more incoherent temporal sense of self [i.e., chronological, cultural, causal, and thematic; (8)]. Note that the majority of these studies are based on borderline PD (BPD). That is, adults with BPD [e.g., (11–13)] construct narrative identities that are less chronologically coherent (i.e., lack orientation and structure) compared to control participants without BPD and people with OCD. Associations have also been found between elevated BPD features in adolescents and less chronological coherent narratives (14). Adults with BPD construct less normative narratives (12) compared to a community sample and people with OCD indicating impoverished cultural coherence in BPD. Adler and colleagues (11) showed negative associations between BPD and the ability to link stories to the person's larger sense of self when compared to a matched control group without BPD and Lind and colleagues found somewhat complex but overly negative causal connections between stories and between stories and the self in people with PD compared to a matched control group without PD pathology (15, 16). Furthermore, prominent narrative themes are present, however problematic, in both adults and adolescents manifesting PD. That is, for those with PD, narrative identity is dominated by themes of thwarted agency (stories of defeat, loss of control, victimization, failures) and strong needs for communion (e.g., belongingness and love) that are unfulfilled [e.g., interpersonal disappointments, betrayal, loneliness; e.g., (11, 15, 16, Lind et al., under review)]. A potential fifth and distinct aspect of coherence, engrained in the attachment literature (17, 18), involves inconsistency (e.g., contradictions) and a lack of plausibility of the narratives (19) and has been associated with, at least, borderline PD [e.g., (20)]. To recap, people manifesting PD show problematic autobiographical reasoning, reflected in diverse types of thwarted coherence. Adolescents manifesting BPD show reduced temporal coherence (14) as well as thematic disturbances related to diminished themes of communion fulfillment and particularly agency (Lind et al., under review) indicating that they struggle with both developmentally early and more sophisticated autobiographical reasoning (8).

## Discussion

### Narrative Identity as a Marker of Self-Function in ICD-11

In ICD-11, aspects of self-function are evaluated on a severity scale when conceptualizing and diagnosing PD. Autobiographical reasoning in narrative identity could be integrated in a similar way, to nuance the coherence aspect of the self-function (see Table 1 for a tentative suggestion). Moving forward, it will be crucial to identify clear cut-off points (i.e., narrative markers) to determine when the autobiographical reasoning converts from being adaptive to mildly-severely disturbed. Lind, Thomsen and colleagues (16) showed that differences in narrative identity predicted group membership (PD vs. control) even after controlling for depressive symptoms—an important first step. Since associations have been shown between inpatient adolescents with PD features and problematic chronological and thematic coherence [(14), Lind et al., under review], autobiographical reasoning could be a critical precursor of full-blown PD together with other identity aspects (22, 23). Because narrative chronology emerges earlier in development than thematic coherence, the former may be used as a more robust narrative marker of early on-set of maladaptive development. Future research should also clearly map out the unique aspects of autobiographical reasoning related to PD and those that might tap into a general *p* factor (24).

**Table 1:** Tentative “Cross Walk” for level of personality functioning focusing on autobiographical reasoning within narrative identity.

**Domains**	**None**	**Mild**	**Moderate**	**Severe**
Diagnosing	The autobiographical reasoning is well-functioning: Stories are chronologically meaningful (e.g., located in time and place), grounded in culture (i.e., inclusion and accurate timing of normative life events). Meaningful links are created between events and between events and the self. These links are predominantly positive. Themes are identified across the lifespan and encompass elevated agency and communion	Some areas of autobiographical reasoning may be affected. For example, stories could be chronologically and culturally coherent, but the causal connections might be predominantly negative or absent in certain parts of the story. Some themes are created across the lifespan, however, they may be characterized by thwarted agency and communion	Multiple areas of autobiographical reasoning are affected. For example, stories might be chronologically coherent (e.g., located in time and place) but less culturally grounded, and with few causal connections that are predominantly negative. The themes that are created are maladaptive and encompass thwarted agency and communion	Autobiographical reasoning is severely disturbed. For example, stories may be severely disorganized and culturally detached. The causal connections are either absent or seriously negative. Thematic connections are either absent or encompass themes of severe thwarted agency and communion
Treatment recommendation	Treatment focusing on repairing autobiographical reasoning is not recommended	Less structured and less intense therapy focusing on the areas of autobiographical reasoning that might be disturbed, for example in a group-based setting or/and by using a flexible narrative repair guide (Thomsen et al., under review)	Moderately structured therapy that incorporates the multiple areas of disturbed autobiographical reasoning in the narrative identity. Incorporating work on narrative identity may also strengthen the alliance that is often threatened at this stage. Any narrative tools should be adjusted to provide moderate structure (Thomsen et al., under review)	Highly structured treatment settings with clear boundaries when working on autobiographical reasoning that is severely disturbed. While a focus on a person's narrative identity may strengthen the working alliance it may be important to work on how autobiographical reasoning is nested within other areas of dysfunction and paying attention to suicidal risks

*Inspired by Bach and First (1), Bach and Simonsen (21), and the ICD-11 Clinical Descriptions and Diagnostic Guidelines for Personality Disorders*.

### Assessment of Autobiographical Reasoning in ICD-11

Narrative identity is typically assessed using the Life Story Interview (25). This semi-structured interview provides rich material on the person's own interpretation on the self and the lived life. Narrative characteristics such as autobiographical reasoning are then assessed using previously validated coding systems. Some crucial aspects must, however, be considered before integrating existing narrative identity assessment in the context of PD. First, the LSI prompts for a prototypical life story and narration in Western countries. It may not adequately capture the normative reasoning and story-telling in other parts of the world, in which some adjustment will be required. Second, autobiographical reasoning covers a niche of personally significant reasoning distinctive from general cognitive difficulties. For example, the disturbances related to autobiographical reasoning in PD were evident even when the control group considered was matched on education [e.g., (11, 15, 16)]. Finally, existing assessment is based on the typically developing personality and, while researchers have started to create coding systems from a clinical stance (e.g., Lind and Dunlop, unpublished coding manual), more is needed to accurately assess personality (dys)functioning within ICD-11. Echoing Hoopwood (26), clinical and basic personality psychologists interact with each other less than they should. This would be a golden opportunity to do so, especially since no official instrument yet exists assessing self- and interpersonal functioning in ICD-11 [e.g., (1, 21)]. In DSM-5's Alternative Model for Personality Disorders [e.g., (27)], identity is evaluated based on the subdomains of self-differentiation, self-esteem, and emotional range and regulation and do not adequately capture the coherent sense of self as it is articulated in ICD-11. As such, adopting existing assessment from DSM-5 may be less than ideal if the temporal aspect of the self should be adequately integrated within forthcoming measurements.

Both written (28) and video-material (29) of the LSI have been used to successfully assess levels of self-functioning in community samples whereas the other-function (e.g., mentalization) were more challenging to assess. Recently, a modified LSI version was used to assess people with BPD's vicarious life story of a parent. That is, the patients were asked to tell their mother or father's life story—to put themselves in their parent's shoes and elaborate and reflect on this story from the parent's perspective (Lind et al., in preparation). Aspects of autobiographical reasoning (i.e., a temporal other- reasoning) and meta-cognition (30), a concept related to mentalization, were successfully assessed. The vicarious perspective (31) could be a fruitful area for future assessment of other-functioning in ICD-11.

### Narrative Identity and Treatment: Implications for ICD-11

Prominent researchers in the field have suggested that the severity of personality functioning can be used as a decision tool to determine the optimal treatment and treatment intensity in a “personalized medicine manner” (22). Given the importance of a coherent identity in ICD-11 [e.g., (1)], the narrative identity seems significant. Several prominent researchers and clinicians [e.g., (32–35)] have emphasized the relevance of integrating narrative identity within psychotherapy. In my own research, I stress the importance of implementing narrative identity within treatment of PD [e.g., (10, 14, 15)] and particularly in the context of the dimensional model (Lind et al., under review, see also 27). Bach and Simonsen (21) further suggested that narrative identity could be a potential mechanism of change in therapy, especially for moderate-to-severe personality disorders in the context of ICD-11. Integrating narrative identity within a treatment setting has several advantages: first, it fits within “personalized/narrative medicine” by emphasizing the importance of a person's unique life, and second, it offers an empathic setting in which the therapist is giving voice and ownership to the patient's story creating a unique and vulnerable window into the person's sense of self nurturing epistemic trust [i.e., the willingness to consider new knowledge as trustworthy and relevant to the self: (36)] and building a working alliance with the patient (21). In Table 1, I offer tentative suggestions on how autobiographical reasoning can be considered within a therapeutic context and dependent on the severity of functioning in ICD-11. Importantly, the origin and underlying mechanisms of reasoning difficulties are complex and can be interpreted and treated from multiple therapeutic approaches. Together with close colleagues, I have recently developed a narrative repair guide to assist people with mental illness in gaining enhanced insight and more adaptive story telling (Thomsen et al., under review). This guide could be helpful in the context of PD and likely in combination with more cognitive evidence-based treatments. However, approaches that perceive the reasoning abnormalities as emanating from splitting-based defense mechanisms [e.g., (17, 37)] or mentalizing difficulties (38) endorsing psychodynamic interventions may also be effective. For example, themes of narrative agency were improved in people manifesting PD after psychodynamic therapy (15). That is, the self has been highlighted as a driver of personality functioning [i.e., Criterion A; (39)]. Strengthening autobiographical reasoning (i.e., the self as author) may scaffold a healthy organization of personality regardless of whether these reasoning challenges are interpreted from a more cognitive or psychodynamic perspective.

## Concluding Remarks

In this paper I stress the importance of considering narrative identity and particularly the role of autobiographical reasoning as a marker of self-(dys)function in the context of ICD-11's dimensional model of PD. I highlight implications for therapy as we dive deeper into the new dimensional era.

## Author Contributions

ML contributed solely to all aspects of this paper.

## Conflict of Interest

The author declares that the research was conducted in the absence of any commercial or financial relationships that could be construed as a potential conflict of interest.
